# Impact of coronary artery calcium scores on cardiovascular risk and preventive therapies: A systematic review and meta-analysis

**DOI:** 10.21542/gcsp.2025.48

**Published:** 2025-10-31

**Authors:** Hussam Al Hennawi, Muhammad Salman Sabri, Muhammad Khuzzaim Khan, Nikhil Duseja, Rida Asim, Robert A. Watson

**Affiliations:** 1 Department of Internal Medicine, Jefferson Abington Hospital, Abington, Pennsylvania, United States; 2 Campus Charité Mitte, Charité — Universitätsmedizin Berlin, Berlin, Germany; 3 Department of Internal Medicine, Karachi Medical and Dental College, Karachi, Pakistan; 4 Department of Internal Medicine, Karachi Medical and Dental College, Karachi, Pakistan; 5 Department of Cardiology, Jefferson Abington Hospital, Abington, Pennsylvania, United States

## Abstract

Introduction: Coronary artery calcium (CAC) scoring is a key noninvasive method for detecting subclinical atherosclerosis and refining cardiovascular risk assessment. It enhances traditional risk models and plays an expanding role in preventive cardiology. This systematic review and meta-analysis aimed to evaluate the relationship between CAC presence and cardiovascular outcomes, as well as its impact on the initiation and continuation of preventive pharmacologic and lifestyle interventions among asymptomatic and symptomatic individuals without known coronary artery disease (CAD).

Methods: Following PRISMA guidelines, a systematic search was conducted through February 2025 across PubMed, Scopus, Google Scholar, Cochrane Central, and ClinicalTrials.gov. Eligible studies included randomized controlled trials and observational research comparing outcomes and behaviors between individuals with a CAC score of zero and those with a score above zero.

Results: Fifty-three observational studies involving 210,672 asymptomatic and 32,477 symptomatic participants were analyzed. Over an average follow-up of 8.6 years, the presence of CAC was significantly associated with increased risks of major adverse cardiovascular events (OR: 5.58), all-cause mortality (OR: 3.90), myocardial infarction (OR: 4.01), and revascularization (OR: 11.90). CAC presence also correlated with greater odds of initiating and continuing preventive therapies such as aspirin, lipid-lowering, and blood pressure medications, as well as adopting healthier lifestyle changes like increased physical activity and improved diet.

Conclusion: CAC is a robust, independent marker of future cardiovascular risk and a strong catalyst for clinical intervention. Its use in risk stratification can enhance personalized prevention strategies in both asymptomatic and symptomatic populations.

## Introduction

Coronary artery disease (CAD) is a progressive condition marked by the accumulation of atherosclerotic plaques within the coronary arteries, often remaining clinically silent until advanced stages. It serves as the primary pathophysiological substrate for coronary heart disease (CHD), or ischemic heart disease (IHD), which includes clinical syndromes such as stable angina, acute coronary syndrome (ACS), and silent myocardial ischemia^1,2^. Given this relationship, we broadly use the term CAD throughout this paper to encompass all manifestations of CHD.

Globally, CAD stands as the leading cause of mortality and Disability-Adjusted Life Years (DALYs), imposing a particularly heavy burden on low- and middle-income countries^1–5^. In 2015, ischemic heart disease accounted for an estimated 8.9 million deaths and 164 million DALYs worldwide^6^. Moreover, survivors of myocardial infarction (MI) continue to face markedly elevated risks of recurrent events and demonstrate an annual mortality rate substantially higher than those without CAD^7–11^.

Optimal management of CAD requires a comprehensive care continuum, beginning with primary prevention and encompassing acute care, accurate diagnosis, revascularization therapies, secondary prevention, and lifelong follow-up (Supplementary File: Table 1). Early risk assessment remains central to preventive cardiology, enabling the timely implementation of risk factor modification strategies such as lifestyle interventions and lipid-lowering therapies^12^. Secondary prevention further aims to avert recurrent MI and related complications, sometimes necessitating invasive procedures like percutaneous coronary intervention (PCI) or coronary artery bypass grafting (CABG).

Despite significant technological and therapeutic advances—including imaging modalities like intravascular ultrasound (IVUS) and optical coherence tomography (OCT)^13,14^, improved PCI techniques, and innovations in automated monitoring systems—CAD remains a formidable public health challenge. This underscores the critical need for improved strategies to detect at-risk individuals before the onset of clinical events^15^.

Coronary artery calcium (CAC) scoring has emerged as a powerful, noninvasive tool for early cardiovascular risk stratification^16^. The identification of calcified coronary plaques using cardiac computed tomography (CT) offers a robust, reproducible predictor of major adverse cardiovascular events (MACE) in asymptomatic populations^17–19^. Beyond augmenting traditional risk factors, CAC scoring provides incremental prognostic information, facilitating the proactive adoption of pharmacological and lifestyle interventions^20–22^.

Extensive evidence supports the prognostic strength of CAC, establishing it as one of the most potent predictors of future CHD and cardiovascular disease (CVD) events^23–25^. Reflecting this, current American College of Cardiology (ACC) and American Heart Association (AHA) guidelines recommend CAC scoring for intermediate-risk individuals when clinical decision-making remains uncertain after traditional risk assessment with pooled cohort equations (PCE)^17,20^.

The Early Identification of Subclinical Atherosclerosis by Noninvasive Imaging Research (EISNER) study demonstrated that visualization of a nonzero CAC score is a strong motivator for initiating preventive pharmacotherapies, including aspirin (ASA), lipid-lowering medications (LLM), and blood pressure-lowering medications (BPLM). These findings emphasize that coronary calcium detection, not merely undergoing imaging, drives preventive behavioral change. Similarly, Gupta et al. confirmed that a nonzero CAC score substantially increases the likelihood of initiating or continuing pharmacologic and lifestyle-based interventions aimed at cardiovascular prevention.

While previous meta-analyses have evaluated the role of CAC in influencing preventive therapies, their scope has been relatively narrow. The present study seeks to provide a more comprehensive and updated synthesis, systematically analyzing cardiovascular outcomes and preventive interventions according to CAC status. We focus on comparing the likelihood of initiating or sustaining pharmacological and lifestyle modifications among individuals with a CAC score greater than zero versus those with a score of zero, in both asymptomatic and symptomatic populations without a prior CAD diagnosis.

## Methods

### Study design and reporting

This systematic review and meta-analysis followed PRISMA (Preferred Reporting Items for Systematic Reviews and Meta-Analyses) guidelines to ensure a transparent and structured approach.

### Data sources and search strategy

A comprehensive literature search was performed across PubMed, Scopus, Google Scholar, Cochrane Central, and ClinicalTrials.gov, covering all available studies from inception to February 2025. We focused on randomized controlled trials and observational studies comparing outcomes in individuals with a coronary artery calcium (CAC) score greater than zero versus those with a CAC score of zero. To ensure inclusivity, we used a combination of free-text search terms and medical subject headings (MeSH) related to CAC and preventive interventions, including aspirin (ASA), blood pressure-lowering medications (BPLM), lipid-lowering medications (LLM), smoking cessation, physical activity, dietary changes, and weight loss. There were no age or study type restrictions, but only studies published in English were included. Further details regarding our search terms, inclusion and exclusion criteria, and language restrictions can be found in the online Supplementary files to this manuscript.

### Study selection

We included studies that examined whether CAC scores influenced subsequent lifestyle changes or medication use aimed at the primary prevention of cardiovascular disease. After duplicate removal, two independent reviewers screened studies based on titles and abstracts to eliminate irrelevant ones. Full-text reviews were then conducted to determine final eligibility. To ensure thoroughness, we also examined the reference lists of selected papers for additional relevant studies. Discussions with the senior author (MKK) resolved any disagreements in study selection.

### Inclusion criteria

• Adults (≥18 years).
• Asymptomatic or symptomatic individuals without known CAD at baseline.
• Studies reporting outcomes according to CAC = 0 vs CAC >0.
• Outcomes: cardiovascular events (MACE, MI, mortality, revascularization), initiation/continuation of pharmacological therapy, or lifestyle modification.
• Study design: observational (prospective, retrospective) or RCTs.
• Studies in English language only.

### Exclusion criteria

• Studies with prior CAD diagnosis at baseline.
• Case reports, reviews, editorials.
• Studies not reporting relevant outcomes or without CAC stratification.
• Poor methodological quality (e.g., incomplete outcome data, NOS <5).

### Data extraction and quality assessment

Two independent reviewers (N.D. and R.A.) extracted data from the included studies. If CAC scores were categorized in a way that did not allow direct comparison between zero and nonzero groups, we contacted the corresponding authors for clarification or additional data. Studies were excluded if the necessary data were unavailable. In cases where multiple papers analyzed the same patient cohort, we extracted data from each publication only if they reported distinct outcomes.

For each study, we recorded baseline characteristics (as detailed in Supplementary File: Table 1) and primary outcomes categorized by CAC score (zero vs. nonzero). We also documented unadjusted and adjusted odds ratios (ORs), noting the statistical models and covariates used for adjustments. Study quality was assessed using the Newcastle-Ottawa Scale.

### Data synthesis and statistical analysis

Our primary goal was to determine the odds ratio (OR) for preventive interventions among individuals with a nonzero CAC score compared to those with a CAC score of zero. We used ORs with 95% confidence intervals (CIs) as the effect size metric. To synthesize the data, we employed the DerSimonian and Laird random-effects model, which accounts for study variations. Unadjusted ORs were pooled across all studies to establish a crude association, while adjusted ORs were recorded from the most comprehensive multivariable models reported by each study.

Heterogeneity was assessed using Cochrane’s Q test and the I^2^ statistic, where an I^2^ value exceeding 50% indicated significant heterogeneity. To evaluate publication bias, we utilized contour-enhanced funnel plots, Egger’s linear regression test, and fail-safe N calculations to estimate the number of missing studies that could nullify statistical significance. We also explored the robustness of our pooled estimates by assessing the impact of imputing missing studies and examining shifts in ORs.

Sensitivity analyses were conducted for outcomes with at least three studies by sequentially excluding one study at a time to assess stability. Additionally, meta-regression was performed to examine whether the length of follow-up influenced study findings, provided at least three studies were available for a given outcome.

All statistical analyses were performed using Review Manager (RevMan) version 5.3 (The Nordic Cochrane Centre, The Cochrane Collaboration, Copenhagen, Denmark). Sensitivity analyses, meta-regression, and publication bias assessments were conducted using Comprehensive Meta-Analysis version 3 (Biostat, Englewood, New Jersey). A two-tailed *p*-value of <0.05 was considered statistically significant.

## Results

**Figure 1.**
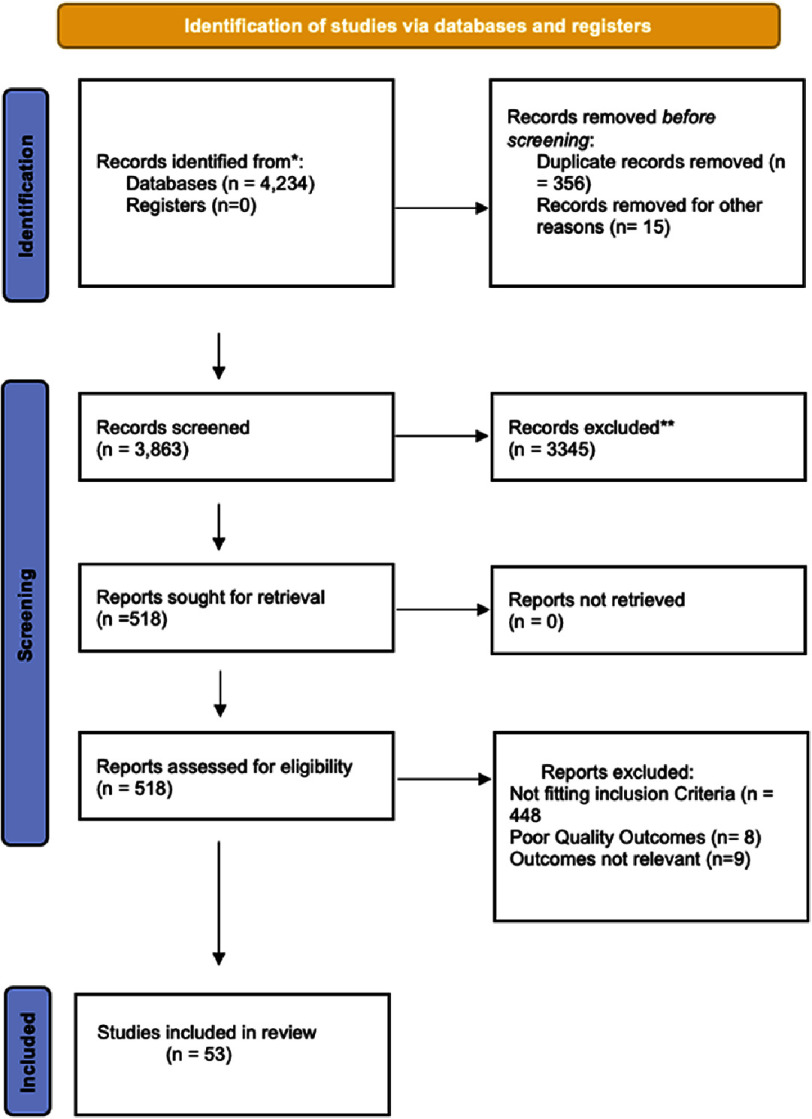
Prisma flow chart.

Our comprehensive search over PubMed, Scopus, Google scholar, Cochrane and Clinicaltrials.gov yielded a total of 4,234 results. This was followed by a rigrous screening process which narrowed the selection to 518 studies based on title and abstract. Finally, a meticulous full-text review against our predefined criteria, 53 studies met the criteria for inclusion in the final analysis. The results have been depicted in PRISMA flow chart in Figure 1.

### Baseline characteristics

Our analysis included 53 observational studies, incorporating data from 210,672 asymptomatic and 32,477 symptomatic patients. The mean follow-up duration across studies was 8.6 years^41–89^.

### Major adverse cardiovascular events

**Figure 2.**
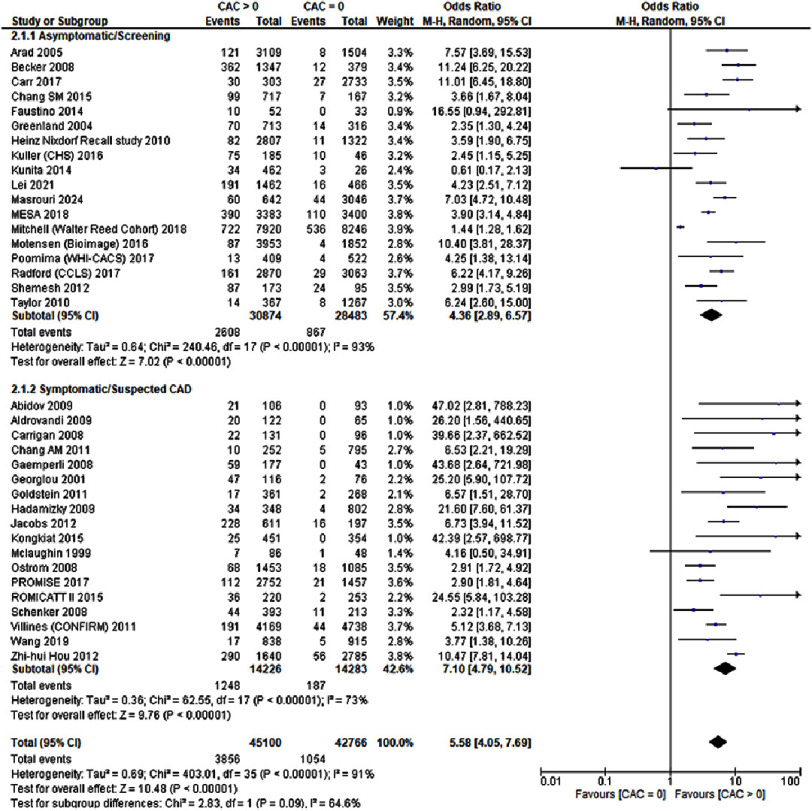
A CAC score greater than zero was associated with an increased risk for MACE.

A total of 36 studies reported the occurrence of MACE amongst those with suspected CAD and those with asymptomatic CAD, including a total of 87,866 patients. We observed that a CAC score greater than zero significantly correlated with an elevated risk of major adverse cardiovascular events (MACE) (OR: 5.58; 95% CI: 4.05–7.69; *P* < 0.00001; I^2^=91%). The observed heterogeneity was significant, and upon the performance of sensitivity analysis, no particular study was revealed to be the source of heterogeneity. These results have been depicted in Figure 2. Meta regression showed no correlation with the follow up time as represented in Figure 11.

### All-cause mortality

**Figure 3.**
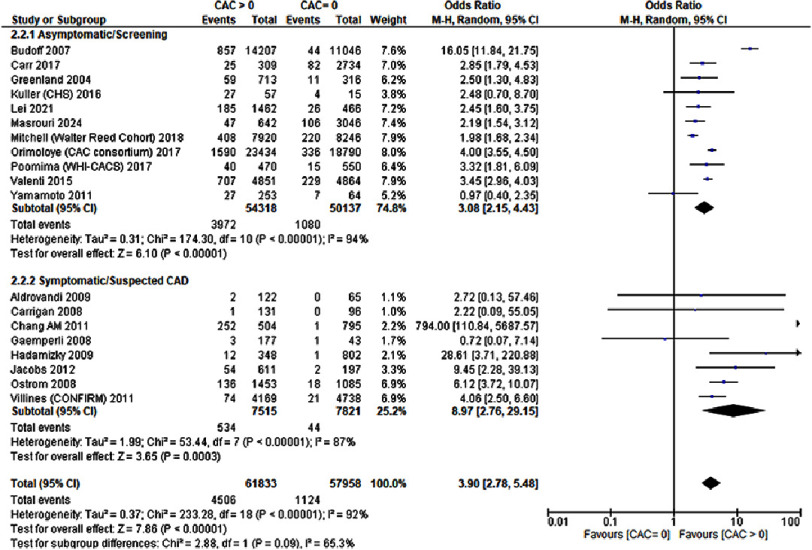
A CAC score greater than zero was associated with an increased risk for all-cause mortality.

A total of 19 studies out of 53 reported the occurrence of all-cause mortality with a total of 119,791 patients included. It was observed that a CAC score greater than zero significantly increased the risk of all-cause mortality (OR: 3.90; 95% CI 2.78–5.48; *P* < 0.00001; I^2^=92%). Significant heterogeneity was observed. Sensitivity analysis revealed that no study was found to be the source of significant heterogeneity. Forest plot in Figure 3 represents the analysis.

**Figure 4.**
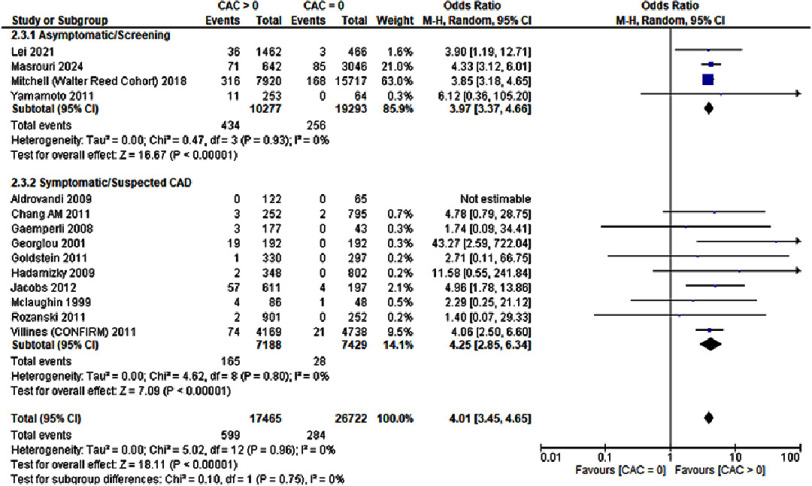
A CAC score greater than zero was associated with an increased risk for myocardial infarction.

### Myocardial infarction (MI)

We evaluated the risk of MI in patients with CAC score = 0 and those with a CAC score greater than 0 as mentioned by a total of 14 studies including 44,187 patients. The analysis revealed that a CAC score greater than 0 was significantly associated with an elevated risk of MI (OR: 4.01; 95% CI: 3.45–4.65; *P* < 0.00001; I^2^= 0%). The results have been depicted in Figure 4.

### Revascularization

**Figure 5.**
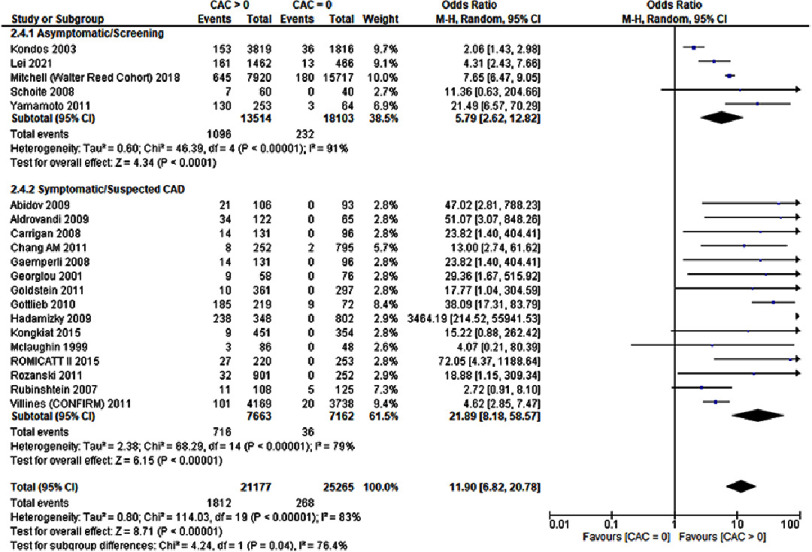
A CAC score greater than zero was associated with an increased risk for revascularization.

The need for revascularization was assessed by 20 studies involving a total of 46,442 patients. The results revealed a significant correlation between CAC score greater than 0 and revascularization (OR: 11.90; 95% CI: 6.82–20.78; *P* < 0.00001; I^2^= 83%). Sensitivity analysis was performed for the significant heterogeneity observed, although no source was obtained. Figure 5 represents the results.

**Figure 6A.**
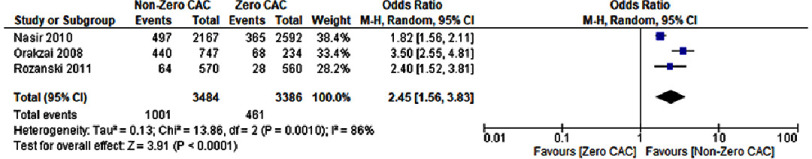
A CAC score greater than zero was associated with an increased likelihood of aspirin initiation.

### Initiation of preventive pharmacological therapy

Patients with a CAC score greater than zero were significantly more likely to initiate preventive pharmacological therapies. The odds of starting aspirin therapy were higher in the nonzero CAC group (OR: 2.45; 95% CI: 1.56–3.83; *P* < 0.0001; I^2^=86%) (Figure 6A). Similarly, lipid-lowering medication initiation was more frequent in this group (OR: 2.44; 95% CI: 1.57–3.79; *P* < 0.0001; I^2^=85%) (Figure 7A, as was the initiation of blood pressure-lowering medications (OR: 1.94; 95% CI: 1.61–2.33; *P* < 0.00001; I^2^= 15%) (Figure 8A).

### Continuation of preventive therapy

Patients with a CAC score greater than zero also exhibited a greater likelihood of continuing preventive therapies. The odds of continuing aspirin therapy were significantly elevated (OR: 1.71; 95% CI: 1.31–2.23; *P* < 0.0001; I^2^=0%) (Figure 6B). Lipid-lowering medications also showed a strong continuation trend (OR: 2.48; 95% CI: 1.53–4.01; *P* = 0.0002; I^2^= 63%) (Figure 7B). However, this analysis did not provide data on the continuation of blood pressure-lowering medications (Figure 8B).

**Figure 6B.**
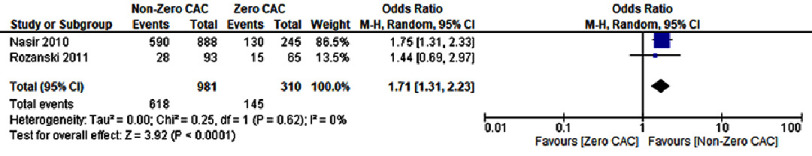
A CAC score greater than zero was associated with an increased likelihood of aspirin continuation.

**Figure 7A.**
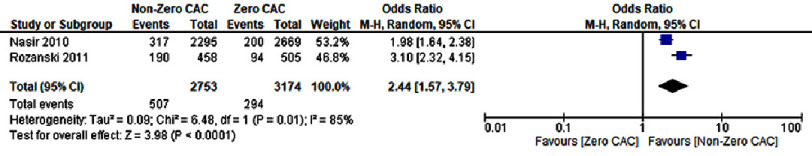
A CAC score greater than zero was associated with an increased likelihood of lipid-lowering medication initiation.

**Figure 7B.**
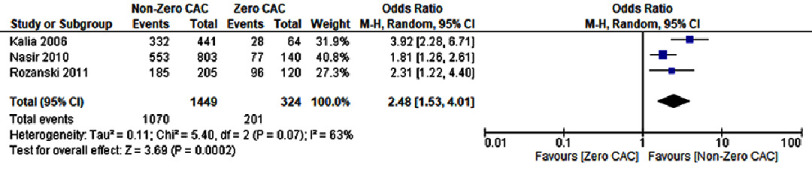
A CAC score greater than zero was associated with an increased likelihood of lipid-lowering medication continuation.

**Figure 8A.**
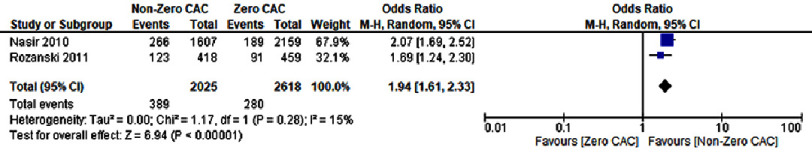
A CAC score greater than zero was associated with an increased likelihood of blood pressure medication initiation.

**Figure 8B.**
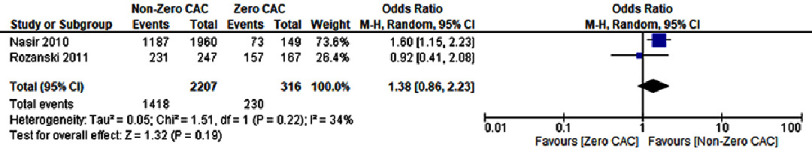
CAC score does not significantly impact blood pressure medication continuation.

**Figure 9.**
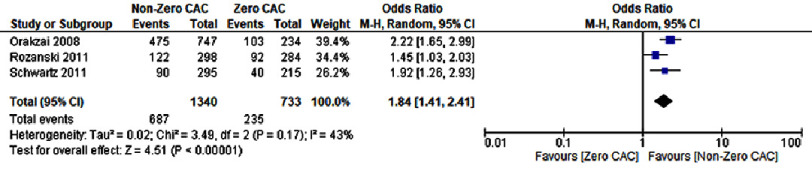
A CAC score greater than zero was associated with increased exercise.

**Figure 10.**
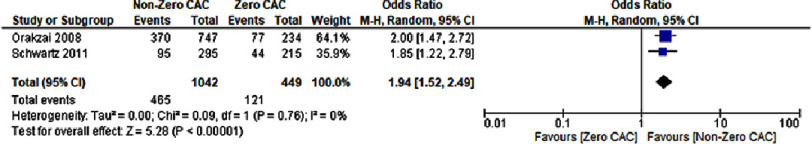
A CAC score greater than zero was associated with dietary change.

**Figure 11.**
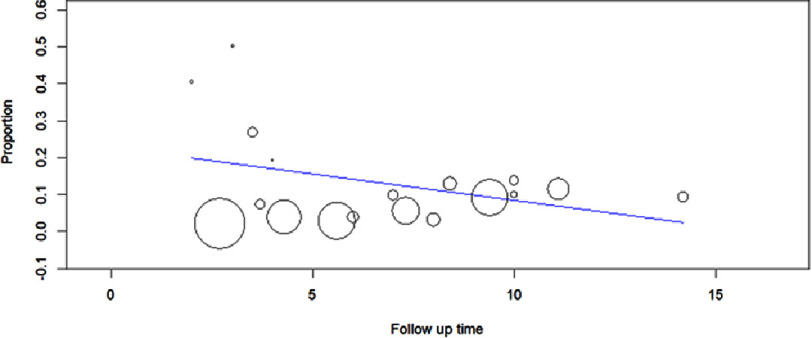
Meta-regression plot for MACE.

### Lifestyle modifications and risk factor control

A CAC score greater than zero was associated with an increased likelihood of lifestyle modifications. Patients were more likely to engage in increased physical activity (OR: 1.84; 95% CI: 1.41–2.41; *P* < 0.00001; I^2^= 43%) and adopt dietary changes (OR: 1.94; 95% CI: 1.52–2.49; *P* < 0.00001; I^2^= 0%) as represented in Figures 9 and 10, respectively.

### Quality assessment

A quality assessment was conducted for the observational studies and randomized controlled trials (RCTs) employing the Newcastle-Ottawa Scale and the Cochrane Risk of Bias 2.0 tool, respectively.

Almost all included studies demonstrated a high methodological rigor and quality level, with a few showing low and moderate quality. The evaluation results are summarized in Supplementary Table 1, providing a snapshot of each study’s strengths and potential limitations. Additionally, Supplementary Figure 2 visually represents the key findings of the quality assessment, offering a clear and concise overview of the methodological integrity and potential biases identified across the included studies.

## Discussion

This systematic review and meta-analysis of 53 observational studies involving over 243,000 individuals reinforces the pivotal role of coronary artery calcium (CAC) in cardiovascular risk stratification and preventive cardiology. The presence of CAC was strongly associated with a markedly increased risk of major adverse cardiovascular events (MACE), including myocardial infarction, all-cause mortality, and the need for revascularization. Beyond traditional risk prediction, CAC status influenced clinical management behaviors; individuals with detectable CAC were significantly more likely to initiate and adhere to preventive therapies, such as aspirin, lipid-lowering, and blood pressure-lowering agents, and to adopt favorable lifestyle modifications, including increased physical activity and dietary changes.

Our findings are consistent with the meta-analysis by Gupta et al., which reported a 2- to 3-fold greater likelihood of preventive pharmacologic initiation and continuation among individuals with CAC^27^. Notably, the impact of CAC on preventive strategies persisted even after adjusting for baseline risk factors, mirroring our observations. Similar evidence from studies utilizing coronary computed tomography angiography further supports the role of plaque detection in catalyzing behavioral change^28,29^.

In alignment with our results, Maseouei et al. demonstrated that CAC scores ≥100 were associated with increased risks of cardiovascular disease (CVD), coronary heart disease (CHD), and mortality, independent of metabolic syndrome (MetS) or diabetes mellitus (DM)^30^. However, while our findings suggest a strong association across all metabolic profiles. Maseouei et al. noted a slightly attenuated risk relationship among diabetic individuals, reflecting the complexity of risk stratification in this subgroup. Nevertheless, both studies affirm that a CAC score of zero correlates with favorable outcomes across metabolic groups.

Consistent with population-based cohort studies^29,31,32^, we found that most asymptomatic adults under age 55 exhibit a CAC score of zero. Yet even among younger individuals aged 30–49 years, the presence of CAC ≥100 significantly elevated CVD and CHD mortality risks^33^. Our findings further highlight that nearly half of diabetic patients aged 40–49 years already possess an intermediate atherosclerotic cardiovascular disease (ASCVD) risk, underscoring the need for enhanced risk stratification. While CAC measurement improves risk prediction, consistent with the 2023 Standards of Care in Diabetes^34^ and the 2023 ESC Guidelines^35^, its clinical utility among asymptomatic diabetic individuals remains an area of ongoing investigation^36–38^.

The debate regarding CAC screening’s cost-effectiveness and potential downstream testing burden persists. However, our findings, supported by data from Rozanski et al.^26^ and economic analyses from MESA^18,39^, suggest that CAC testing does not substantially escalate procedural costs and remains cost-effective for intermediate-risk individuals.

CAC screening is most useful in intermediate-risk patients where treatment decisions are uncertain after pooled cohort equations (PCE). It further helps to provide prognostic information beyond traditional risk factors. CAC scoring can be used to guide statin initiation, aspirin use and lifestyle modification^90^. Comparing the cost-effectiveness, CAC testing has shown to be more cost effective as a result of improved targeting of statin therapy and avoiding lifelong medication. MESA analysis found CAC guided statin allocation to be more efficient and cost-effective compared with universal statin use^19^. According to the existing literature, visualization of CAC strongly motivates patients to initiate and adhere to preventive pharmcotherapies and lifestyle changes. However, possible negative psychological effects include anxiety, fear, or unnecessary worry in patients with high score^22^.

Although concerns regarding radiation exposure and psychological impacts exist, particularly in younger populations, our findings corroborate evidence that CAC detection offers substantial predictive value even among adults traditionally considered low risk^40^. This data supports the evolving paradigm wherein biological atherosclerotic burden, rather than chronological age alone, guides personalized preventive strategies.

## Limitations

Several limitations should be considered when interpreting our findings. First, the included studies were observational, which may introduce inherent biases such as residual confounding. Second, heterogeneity existed across study populations, imaging techniques, and follow-up durations, although random-effects modeling mitigated this impact. Third, while most studies adjusted for traditional cardiovascular risk factors, variations in multivariable adjustments may influence pooled estimates.

Additionally, data on the continuation of blood pressure-lowering medications were limited, restricting comprehensive evaluation in this category. Furthermore, although CAC is a validated predictor of risk, it does not capture non-calcified plaque burden, particularly relevant in subpopulations such as individuals with diabetes. Many of the studies reported CAC score in binary form (0 vs >0). Using this cutoff helped us maximize the number of studies selected, increasing the generalizability. Furthermore, CAC >0 is the most consistently reported threshold in the literature, whereas higher values such as 100, 400 were inconsistently defined across cohorts, bringing about a challenge in the pooled analysis. Another limitation to be noted is the use of observational studies, which limits casual inference. The association observed between CAC presence and preventive therapy initiation or lifestyle changes may reflect residual confounding (e.g older age, higher baselines risk, physicians prescribing behavior) rather than a direct casual effect of CAC visualization. Thus, our findings should be interpreted as associations rather than proof of casualty.

The observed heterogeneity particularly for outcomes like revasularization (I^2^=83%) and MACE (I^2^ = 91%), likely reflect differences in study populations, follow-up durations, and definitions of clinical endpoint. Although, the direction of effect consistently favored higher risk in individuals with CAC score >0. Therefore, the findings remain clinically impactful, suggesting CAC as a strong prognostic tool across various settings. Additionally, outcomes with low heterogeneity eg (MI= 0%), further reinforce the robustness of these associations.

## Conclusion

Our meta-analysis underscores the critical role of coronary artery calcium scoring in preventive cardiology. CAC presence robustly stratifies cardiovascular risk and serves as an actionable marker that promotes the initiation and adherence to preventive pharmacologic therapies and lifestyle modifications. These findings reinforce CAC’s clinical utility in predicting adverse cardiovascular outcomes and actively guiding risk-reducing interventions. While the predictive value of CAC is broadly supported, nuanced considerations are necessary in specific subgroups such as diabetic individuals. Moreover, although concerns regarding cost and downstream testing remain, emerging evidence supports the cost-effectiveness and clinical benefit of CAC-guided prevention strategies. Future research should continue to refine the role of CAC in individualized cardiovascular risk management, particularly in younger and high-risk populations.

## Conflicts of interest

The authors declare no competing interests.

## Funding

The authors declare no funding support.

## Data Availability Statement

Publicly available data was used.

## Author statement

**Hussam Al Hennawi:** Conceptualization, Methodology, Writing- Original draft preparation

**Muhammad Khuzzaim Khan:** Data curation, Writing- Original draft preparation.

**Muhammad Salman Sabri:** Visualization, Investigation.

**Robert A. Watson:** Supervision
